# ﻿New species of *Hydnotrya* (Ascomycota, Pezizomycetes) from southwestern China with notes on morphological characteristics of 17 species of *Hydnotrya*

**DOI:** 10.3897/mycokeys.100.106709

**Published:** 2023-11-13

**Authors:** Lin Li, Shan-Ping Wan, Yun Wang, Naritsada Thongklang, Song-Ming Tang, Zong-Long Luo, Shu-Hong Li

**Affiliations:** 1 College of Agriculture and Biological Science, Dali University, Dali 671003, Yunnan, China Dali University Dali China; 2 School of Science, Mae Fah Luang University, Chiang Rai 57100, Thailand Mae Fah Luang University Chiang Rai China; 3 Center of Excellence in Fungal Research, Mae Fah Luang University, Chiang Rai, 57100, Thailand Yunnan Agricultural University Kunming China; 4 College of Resources and Environment, Yunnan Agricultural University, Kunming 650201, Yunnan, China New Zealand Institute for Crop and Food Research Limited, Invermay Agricultural Centre Mosgiel New Zealand; 5 New Zealand Institute for Crop and Food Research Limited, Invermay Agricultural Centre, Private Bag 50034, Mosgiel, New Zealand Mae Fah Luang University Chiang Rai Thailand; 6 Biotechnology and Germplasm Resources Institute, Yunnan Academy of Agricultural Sciences, Kunming 650223, Yunnan, China Biotechnology and Germplasm Resources Institute, Yunnan Academy of Agricultural Sciences Kunming China

**Keywords:** Discinaceae, hypogeous fungi, ITS, morphological diversity, taxonomy

## Abstract

More specimens of *Hydnotrya* have been collected from southwestern China in recent years. Morphological and molecular analyses showed that they belonged to three species of *Hydnotrya*, of which two are new to science, *H.oblongispora* and *H.zayuensis*. The third one was *H.laojunshanensis*, previously reported in 2013. The new species are described, and their relationship to other species of *Hydnotrya* is discussed. *H.laojunshanensis* is re-described in more detail. The main morphological characters of 17 species of *Hydnotrya* are compared and a key to them is provided as well.

## ﻿Introduction

*Hydnotrya* Berk. & Broome is a genus of hypogeous fungi belonging to Pezizomycetes, Ascomycota. It was placed in the family Helvellaceae by Spooner (1992) and Abbott and Currah (1997) but based on the recent molecular analyses it has been shifted into the family Discinaceae (O’Donnell et al. 1997; Hansen and Pfister 2006; Tedersoo et al. 2006; Læssøe and Hansen 2007; Wang et al. 2023). Their ascomata are hollow to convoluted with simple or folded chambers, even nearly solid, lined with recognizable hymenium. *Hydnotrya* species usually forms a symbiotic relationship with both conifer and broadleaf trees and are distributed throughout the northern hemisphere (Trappe 1975; Spooner 1992; Trappe and Castellano 2000; Stielow et al. 2010; Xu et al. 2018; Slavova et al. 2021). There are 22 names listed in the Index Fungorum online database (http://www.indexfungorum.org/Names/Names.asp). However, among them, the species *H.jurana* Quél. and *H.carnea* (Corda) Zobel was synonymized with *H.tulasnei* (Berk.) Berk. & Broome (Gilkey 1954; Trappe 1969), *H.ploettneriana* (Henn.) Hawker, *H.yukonensis* Gilkey and *H.dysodes* Kirschstein with *H.michaelis* (E. Fisch.) Trappe (Soehner 1942; Trappe 1975), and *H.convoluta* (McAlpine) McLennan was renamed as *Pezizajactata* Burds. & Korf (Burdsall-Jr 1968), *H.ellipsospora* Gilkey combined as *P.ellipsospora* (Gilkey) Trappe (Trappe 1979). To date, there are 15 accepted species remaining in the genus *Hydnotrya*.

To date, nine *Hydnotrya* species have been reported in China: *H.cerebriformis* in Shanxi and Xinjiang, *H.cubispora* in Tibet, *H.michaelis*, *H.tulasnei* and *H.brunneospora* in Jilin (Tao and Liu 1989; Zhang 1991; Xu 2000; Xu et al. 2018), *H.laojunshanensis* and *H.badia* in Yunnan (Li et al. 2013), *H.nigricans* in Sichuan, *H.puberula* in Yunnan and Jilin (Xu et al. 2018).

Over the past two years, more *Hydnotrya* specimens have been collected in southwest China. Based on the morphological and molecular analyses, two new species were detected and described: *H.oblongispora* and *H.zayuensis*. Their relationships with other known *Hydnotrya* species are discussed and a more detailed supplementary description is given to another species *H.laojunshanensis*, previously found in Yunnan. Additionally, the main morphological characteristics of 15 species of *Hydnotrya* are listed and a key to the species of the genus is provided.

## ﻿Materials and methods

The specimens were collected from Yunnan and Tibet, China. The type and other studied specimens were deposited at the Biological Science Museum of Dali University (BMDLU) and HKAS (Herbarium of Kunming Institute of Botany, Academy Sinica), China.

Descriptions of microscopic and macroscopic characters were based on specimens (BMDLU L20069, L20067, L21197, L21211, L21212, L21215, L21217, L22024, L22027, and HKAS95802) following the methods of Kumar et al. (2017) and Truong et al. (2017). The sections were made with a razorblade by hand, mounted in a 5% KOH solution or water, and then stained with a cotton blue or lactophenol solution. The sections were observed under an Olympus BH-2 microscope. Key colors were obtained from Kornerup and Wanscher (1978).

Total genomic DNA was extracted from the specimen using the OMEGA Plant Genomic DNA Kit. The internal transcribed spacer (ITS) rDNA region was amplified with PCR primers ITS1F and ITS4 (White et al. 1990; Gardes and Bruns 1993; Truong et al. 2017). The large subunit nuclear ribosomal DNA (LSU) region was amplified with the PCR primers LROR and LR5 (Vilgalys and Hester 1990). PCR reactions were performed on a BIO-RAD C1000TM instrument. Thermal cycles with the following settings: initial denaturation for 5 min at 94 °C, followed by 32 cycles of 40 s denaturation at 94 °C, annealing at 56 °C for 40 s for ITS, and 52 °C for 30 s for LSU, extension for 1 min at 72 °C, and final extension at 72 °C for 10 min. The PCR products were verified on 1% agarose electrophoresis gels stained with ethidium bromide. The purification and sequencing of the PCR products was conducted by Sangon Biotech Limited Company (Shanghai, China).

**Figure 1.**
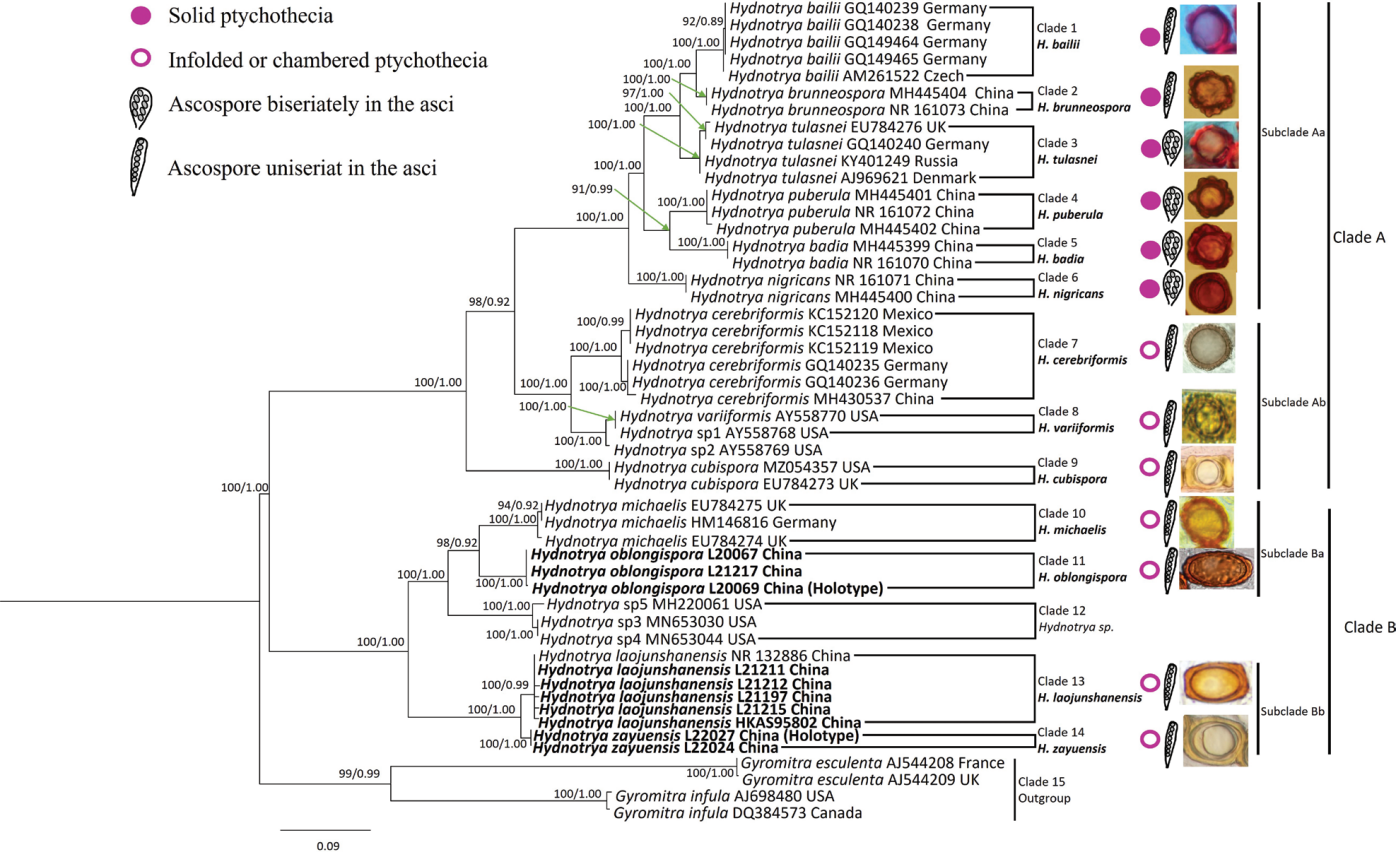
Phylogeny derived from a maximum likelihood (ML) analysis of the nrDNA-ITS sequences from *Hydnotrya* species, using *Gyromitraesculenta* and *G.infula* as outgroup. Values next to nodes reflect, maximum likelihood bootstrap support values (BS), left, and Bayesian posterior probabilities (PP), right. Names of novel species and samples with newly generated sequences in bold. Symbols by taxon names indicate specific fruiting body types, the arrangement of the ascospores in the ascus and ascospore appearance.

ITS was used for the analysis of *Hydnotrya* species diversity in this study because ITS appears as a useful locus for the delimitation of *Hydnotrya* species. 46 ITS sequences from NCBI and this study representing 14 species of *Hydnotrya* (Table 1), including *Gyromitrainfula* (Schaeff.) Quél. and *Gyromitraesculenta* Pers. ex Fr. as outgroups (Fig. 1). All *Hydnotrya*ITS sequences were extracted with an ascoma. Sequences of *Hydnotrya* species generated in this study were submitted to the GenBank database. We first used the Basic Local Alignment Search Tool for the GenBank database to recheck whether the newly generated sequences were amplified DNA from contaminant or not and examine clusters with closely related sequences. DNA sequences were retrieved and assembled using SeqMan. Sequence alignments were aligned using MAFFT version 7 (Katoh and Standley 2013), ITS gene was analyzed using BioEdit v. 7 (Hall 2007) Maximum Likelihood (ML) analysis was performed using RAxML-HPC2 v. 8.2.12 (Stamatakis 2014) as implemented on the Cipres portal (Miller et al. 2011), with the GTR+G+I model and 1,000 rapid bootstrap (BS) replicates for all genes. A reciprocal 70% bootstrap support approach was used to check for conflicts between the tree topologies from individual genes. As the topology of the ML tree and the Bayesian tree are similar, the ITS1, ITS2, and 5.8s sequences were combined using SequenceMatrix (Vaidya et al. 2011), partitioned phylogenetic analyses. For Bayesian Inference (BI), the best substitution model for each partition was determined by MrModeltest 2.2 (Nylander et al. 2004). The result suggested that ITS1: JC+I, 5.8S: GTR+G+I, ITS2: K80+I+G. Bayesian analysis was performed using MrBayes ver. 3.2.7a (Ronquist et al. 2011) on the Cipres (Miller et al. 2011), four parallel runs, were performed for 10 million generations sampling every 100^th^ generation for the single gene trees. Parameter convergence > 200 was verified in Tracer v. 1.7 (Rambaut et al. 2018). The phylogenetic clade was strongly supported if the bootstrap support value (BS) was ≥ 70% and/or a posterior probability (PP) <0.01.

**Table 1.** Taxa information and GenBank accession numbers of the sequences used in this study. The newly generated sequences are in bold.

Species name	Voucher	Origin	GenBank No.	Reference
*Gyromitraesculenta*	Gyr3	France	AJ544208	Kellner et al. (2007)
*Gyromitraesculenta*	m954	UK	AJ544209	Kellner et al. (2007)
*Gyromitrainfula*	UBC F15196	Canada	DQ384573	GenBank
*Gyromitrainfula*	Vellinga GLM	USA	AJ698480	Kellner et al. (2007)
*Hydnotryabadia*	BJTC:FAN270	China	NR_161070	Yu et al. (2018)
*Hydnotryabadia*	BJTC:FAN270	China	MH445399	Yu et al. (2018)
*Hydnotryabailii*	PRM 902032	Czech	AM261522	Stielow (2010)
*Hydnotryabailii*	P.Reil_2	Germany	GQ140239	Stielow (2010)
*Hydnotryabailii*	P.Reil	Germany	GQ140238	Stielow (2010)
*Hydnotryabailii*	997	Germany	GQ149465	Stielow (2010)
*Hydnotryabailii*	979	Germany	GQ149464	Stielow (2010)
*Hydnotryabrunneospora*	HMAS 97138	China	NR_161073	Yu et al. (2018)
*Hydnotryabrunneospora*	HMAS 97138	China	MH445404	Yu et al. (2018)
*Hydnotryacerebriformis*	89_A12_Stielow	Germany	GQ140236	Stielow (2010)
*Hydnotryacerebriformis*	87_G11_Stielow	Germany	GQ140235	Stielow (2010)
*Hydnotryacerebriformis*	BJTC:FAN647	China	MH430537	Yu et al. (2018)
*Hydnotryacerebriformis*	GO-2010-097	Mexico	KC152120	Piña-Páez et al. (2017)
*Hydnotryacerebriformis*	GO-2009-455	Mexico	KC152118	Piña-Páez et al. (2017)
*Hydnotryacerebriformis*	GO-2009-242	Mexico	KC152119	Piña-Páez et al. (2017)
*Hydnotryacubispora*	SAT-13-273-01	USA	MZ054357	GenBank
*Hydnotryacubispora*	K(M)104976	UK	EU784273	Brock et al. (2009)
*Hydnotryalaojunshanensis*	YAAS L2425	China	NR_132886	Li et al. (2013)
***Hydnotryalaojunshanensis***	**BMDLU L21211**	**China**	**ON982580**	**This study**
***Hydnotryalaojunshanensis***	**BMDLU L21212**	**China**	**ON982593**	**This study**
***Hydnotryalaojunshanensis***	**BMDLU L21215**	**China**	**ON982594**	**This study**
***Hydnotryalaojunshanensis***	**BMDLU L21197**	**China**	**ON982592**	**This study**
***Hydnotryalaojunshanensis***	**HKAS95802**	**China**	**OP908303**	**This study**
*Hydnotryamichaelis*	K(M)61643	UK	EU784275	Brock et al. 2009
*Hydnotryamichaelis*	K(M)38647	UK	EU784274	Brock et al. 2009
*Hydnotryamichaelis*	6463-307EMC	Germany	HM146816	Cox et al. 2010
*Hydnotryanigricans*	BJTC:FAN349	China	NR_161071	Yu et al. 2018
*Hydnotryanigricans*	BJTC:FAN349	China	MH445400	Yu et al. 2018
***Hydnotryaoblongispora***	**BMDLU L20067**	**China**	**OM232075**	**This study**
***Hydnotryaoblongispora***	**BMDLU L20069(Holotype)**	**China**	**OM232079**	**This study**
***Hydnotryaoblongispora***	**BMDLU L21217**	**China**	**OM232084**	**This study**
*Hydnotryapuberula*	BJTC:FAN721	China	NR_161072	Yu et al. 2018
*Hydnotryapuberula*	BJTC:FAN721	China	MH445401	Yu et al. 2018
*Hydnotryapuberula*	HMAS96758	China	MH445402	Yu et al. 2018
*Hydnotryatulasnei*	K(M)99871	UK	EU784276	Brock et al. 2009
*Hydnotryatulasnei*	Berk. & Broome C34659	Denmark	AJ969621	Tedersoo et al. 2006
*Hydnotryatulasnei*	IT8	Germany	GQ140240	Stielow 2010
*Hydnotryatulasnei*	605040	Russia	KY401249	GenBank
*Hydnotryavariiformis*	TK1615	USA	AY558770	Izzo et al. 2005
***Hydnotryazayuensis***	**BMDLU L22024**	**China**	**OP908304**	**This study**
***Hydnotryazayuensis***	**BMDLU L22027 (Holotype)**	**China**	**OP908305**	**This study**
*Hydnotrya* sp1.	SNF160	USA	AY558768	Izzo et al. 2005
*Hydnotrya* sp2.	SNF82	USA	AY558769	Izzo et al. 2005
*Hydnotrya* sp3.	JT19176	USA	MN653030	GenBank
*Hydnotrya* sp4.	JT19085	USA	MN653044	GenBank
*Hydnotrya* sp5.	JLF2015	USA	MH220061	GenBank

## ﻿Results

### ﻿Phylogenetic analysis

The ML and Bayesian analyses of the 50 ITS sequences, are shown in Fig. 1 with associated bootstrap supports for branches.

In the phylogenetic tree, the 46 ITS sequences from *Hydnotrya* ascomata revealed the phylogenetic relationship of 14 species: Clade 1 includes 5 sequences of *H.bailii* from Europe. Clade 2 includes 2 sequences of *H.brunneospora* from China. Clade 3 includes 4 sequences of *H.tulasnei* from Europe. Clade 4 includes 3 sequences of *H.puberula* from China. Clade 5 includes 2 sequences of *H.badia* from China. Clade 6 includes 2 sequences of *H.nigricans* from China. Clade 7 includes 6 sequences of *H.cerebriformis* from Germany, China, and Mexico; two other distinct clades were revealed, one comprising Eurasian specimens, and the other comprising specimens from Mexico, which is probably because these specimens, respectively, are from Holarctic and Neotropical regions. Clade 8 includes 3 sequences of *H.variiformis* from the USA. Clade 9 includes 2 sequences of *H.cubispora* from the UK and USA. Clade 10 includes 3 sequences of *H.michaelis* from Europe. Clade 11 includes 3 sequences of new species, *H.oblongispora* from China. Clade 12 includes 3 sequences of *Hydnotrya sp.* from the USA. They may be new species from North America that have not yet been reported. Clade 13 includes 6 sequences of *H.laojunshanensis* from China. When the latter was reported, only one specimen was found, and many more were collected over the past few years, so new DNA sequences of *H.laojunshanensis* were added. Clade 14 includes 2 sequences of a new species, *H.zayuensis* from China. The phylogenetic analysis shows that the new species are distinct from other *Hydnotrya* species. In addition to the ITS sequences used in this phylogenetic analysis, the LSU sequences were amplified from the newly supplemented specimens in this study and uploaded to NCBI for future study.

Based on the ITS locus, two major monophyletic lineages are presented, showing a strong sister relationship (BS=100%; PP = 1.0). They are Clade A (including Clade 1–9) and Clade B (include Clade 10–14) respectively. The species included in these two phylogenetic morphologically share commonalities and uniqueness.

### ﻿Taxonomy

#### ﻿ Hydnotrya oblongispora

Taxon classificationFungiPezizalesDiscinaceae

L. Li & S.H. Li, sp. nov.

2822CD1C-B82E-5890-AD53-90CC02DD02B9

846735

Plate 1

##### Diagnosis.

Differs from other species in the genus *Hydnotrya* by its nearly single-chambered ascomata and long ellipsoidal ascospores.

##### Etymology.

oblongispora, refers to the long ellipsoidal ascospores.

##### Holotype.

China, Yunnan, Lijiang (26°37.00'N, 99°42.00'E), alt. 3737 m, in the forest of *Abiesforrestii* Coltm.-Rog, 12 August 2020, Lin Li, BMDLU L20069.

##### Description.

***Ascomata*** irregularly globose, 1.0–2.5 cm in diameter when fresh, smooth, sometimes gently folded inward, surface light khaki (4C5) to reddish brown (8D8); nearly single-chambered with a primary apical opening up to 0.2–0.8 cm in diameter, sometimes the opening is just an almost closed seam, white fluffy inside cavity. Elastic and crisp. No special smell was noticed.

***Peridium*** two-layered, 280–340 µm thick, outer layer 80–100 µm thick, composed of light brown (6D8) ellipsoidal or irregular cells, with a red brown (6E8) pigment deposited on the outermost cells; inner layer, 200–240 µm thick, consists of hyaline interwoven hyphae. Gleba chamber hollow, lined with a milky white (4B2) hymenium, hymenial surface fluffy. *Asci* cylindrical, 102.5–138.5 × 13.0–25.5 µm, 8-spored, thin-walled, narrowed into a long stalk (20–35 μm) at the base, without croziers, arranged in a palisade. *Ascospore* strictly uniseriate, long ellipsoidal, (20.0–) 26.5–39.0 × (9.5–) 11.0–21.5 μm, Q = 2.0±0.03, hyaline when immature, golden yellow (5B7) when mature, with a thickened exosporium, surface pitted. *Paraphyses* hyaline, straight stick shape, 2.5–5 µm in diam, septate, exceeding the asci by 60–70 µm.

##### Ecology and distribution.

Hypogeous, solitary, or in groups in soil, under *A.forrestii* mixed with shrubs of *Rhododendron* spp., fruiting from late summer to early autumn. Known only from Yunnan Province, China.

##### Additional specimens examined.

China, Yunnan Province, Lijiang, Jiuhe, (26°38.00'N, 99°42.00'E), alt. 3946 m, in the forest of *A.forrestii*, 12.Aug.2020, Lin Li (BMDLU L20067. GenBank: ITS = OM232075, LSU = ON982626); same locality, 19.Sept.2021, Lin Li (BMDLU L21217. GenBank: ITS = OM232084, LSU = ON982625).

##### Notes.

*H.oblongispora* is characterized by its mostly simple-chambered ascomata and golden yellow long-ellipsoid ascospores, especially with pitted surfaces, which differ from all other species of *Hydnotrya.* Molecular analysis also shows that *H.oblongispora* is distinct from other *Hydnotrya* species, although it is closely related to *H.michaelis*. However, *H.michaelis* has convoluted, lobed ascomata and broadly ellipsoid spores with warty ascospores, which differ from this new species.

**Plate 1.**
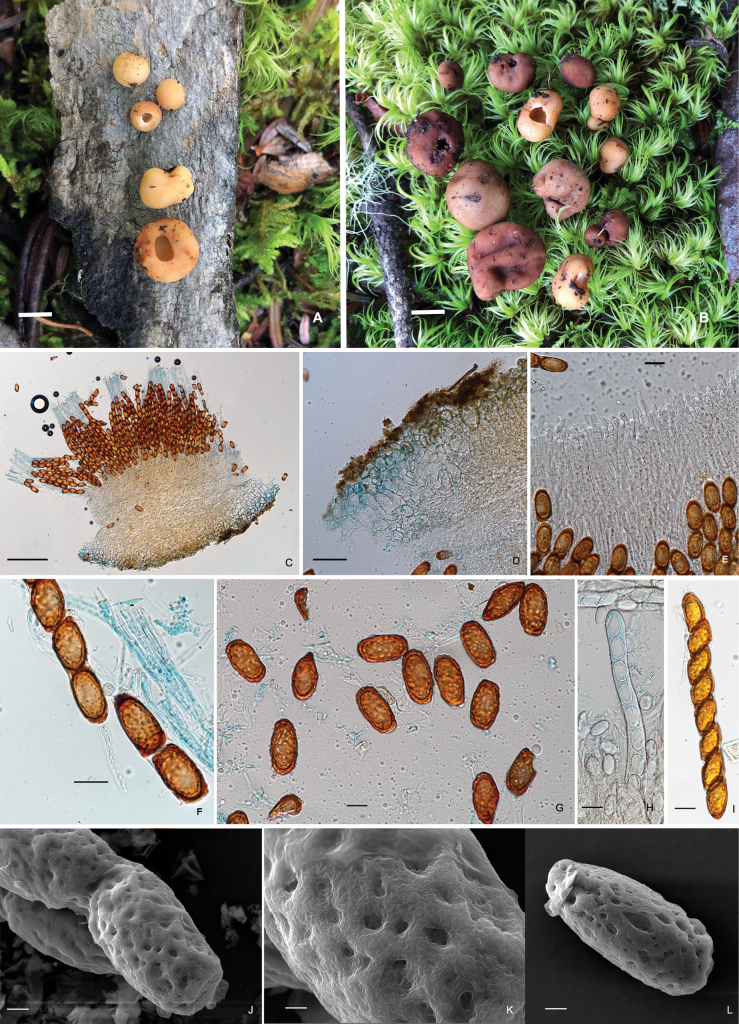
*Hydnotryaoblongispora***A** young sarcomata **B** mature ascomata with different openings **C** a piece of section of the ascomata in lactophenol cotton blue **D** a peridium section in lactophenol cotton blue **E** a section of paraphyses in 5% KOH **F** a base of asci in lactophenol cotton blue **G** ascospores released from the ascus **H** asci in lactophenol cotton blue **I** an ascus with 8 ascospores **J–L** ascospores under SEM. Scale bars: 1 cm (**A, B**); 100 μm (**C**); 50 μm (**D**); 10 μm (**E–I**); 5 μm (**J, L**); 2 μm (**K**).

#### ﻿ Hydnotrya zayuensis

Taxon classificationFungiPezizalesDiscinaceae

L. Li & S.H. Li, sp. nov.

5F9696A3-8A83-5D01-B82D-C06FF654CC96

846736

Plate 2

##### Diagnosis.

Differs from all other species in *Hydnotrya* by its almost single-chambered ascomata, light golden yellow ellipsoidal ascospores.

##### Etomology.

zayuensis from Latin, referring to the type locality.

##### Holotype.

China, Tibet, Zayu (28°35.00'N, 98°06.00'E), alt. 3770 m, in a forest of *Abies* sp., 11 August 2022, Lin Li BMDLU L22027.

##### Description.

***Ascomata*** irregularly globose, 1.5–2.5cm in diameter when fresh, smooth, convoluted, almost single-chambered with a primary apical opening, sometimes the opening nearly closed like a seam, white fluffy inside, surface cinnamon (5E8); shrunken, becoming fuzzy when dried, although there are no protruding hyphae cells from the outermost layer of the peridium. Elastic to crisp. No special smell was noticed.

***Peridium*** two-layered, 180–250 µm thick, outer layer 40–80 µm thick, composed of ellipsoid or irregular cells, which grow larger toward the surface, with a yellow brown (4C5) pigment deposited on the outermost cells; inner layer, 110–160 µm thick, consisting of hyaline parallel interwoven hyphae. Gleba chamber hollow, lined with off-white (1A2) hymenium when immature; two-layered when mature, the outer layer golden brown (5C7), the inner layer yellowish to whitish (4A2), hymenial surface fluffy. *Asci* cylindrical, 118.5–130.5 × 15.0–22.5 µm, 8-spored, thin-walled, narrowed into a long stalk (20–40 μm) at the base, without croziers, arranged in a palisade. *Ascospore* strictly uniseriate, ellipsoid (shape including the thickened exosporium), (17–)20–30.5 × 15.5–18.0 μm, Q = 1.5 ± 0.16, hyaline, exosporium thin when immature, surface roughness, and looks crumbly, golden yellow (4B8) when mature. *Paraphyses* hyaline, straight stick shape, 1.5–2.5 µm in diam, septate, apical slightly inflated, exceeding the asci by 120–160 µm.

**Plate 2.**
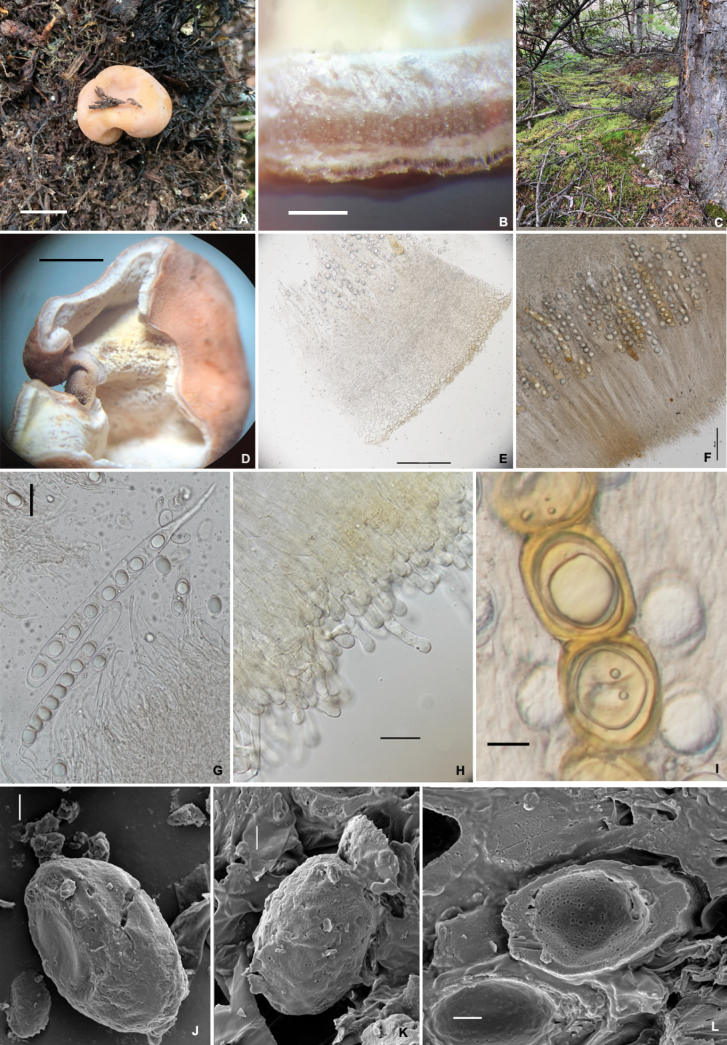
*Hydnotryazayuensis***A** ascomata **B** section of ascomata, with hymenium-lined chambers **C** habitat **D** inner surface of ascomata **E** peridium in 5% KOH **F** hymenium **G** asci in 5% KOH **H** paraphyses **I** ascospores in 5% KOH **J–L** ascospores under SEM (L. SEM of a single ascospore cut in half). Scale bars: 1cm (**A**); 1 mm (**B**); 0.5cm (**D**); 100 μm (**E**); 50 μm (**F**); 20 μm (**G**); 10 μm (**H**); 10 μm (**I**); 5 μm (**J–L**).

##### Ecology and distribution.

Hypogeous, solitary in the humus under *Abies* sp. mixed with shrubs of *Rhododendron* spp. Fruiting in summer, from July to September. Known only from Zayu, Tibet, China.

##### Additional specimen examined.

China, Tibet, Zayu, 28°47.00'N, 98°21.00'E, alt. 3840 m, in a forest of *Abies* sp., 15.July.2022, Shucheng He (BMDLU L22024. GenBank: ITS = OP908304, LSU = OP908301).

##### Notes.

Morphologically, *H.zayuensis* is similar to *H.laojunshanensis.* However, *H.zayuensis* has much smaller ascospores, and a thinner peridium, as well as lighter colored ascomata. Molecular analysis showed that *H.zayuensis* is distinct from *H.laojunshanensis* and other species of *Hydnotrya*.

#### ﻿ Hydnotrya laojunshanensis

Taxon classificationFungiPezizalesDiscinaceae

L. Li, D.Q. Zhou & Y.C. Zhao 2013

576322B7-DB9F-52B6-B2F8-1289554ECEDD

803968

Plate 3

##### Description.

***Ascomata*** irregularly globose, 1.0–3.0 cm in diameter when fresh, brownish orange (6C8), smooth, mostly single-chambered with a primary apical opening to 0.1–0.5 cm in diameter, the opening rarely narrowing into a slit, sometimes folded forming few channels, lined with white fluffy hymenium. Elastic to crisp. No special smell was noticed.

***Peridium*** two-layered, 350–570 µm thick, outer layer 160–200 µm thick, composed of light brown (6E8) angular or irregular cells, inner layer, 220–350 µm thick, consisting of hyaline interwoven hyphae. Gleba chamber hollow, lined with off-white (1A2) hymenium when immature; two-layered when mature, the outer layer orange (6B8), the inner layer yellowish to whitish (4A2), hymenial surface fluffy. *Asci* cylindrical, 331.5–390.5 × 25.5–35.5 µm, 8-spored, thin-walled, narrowed at the base into a long stalk (30–50 μm), without croziers, arranged in a palisade. *Ascospore* strictly uniseriate, ellipsoid (excluding the thickened exosporium), rectangular (with the exosporium), (26.5–)33.0–50.5 × (15.5–)20.5–35.5(–38.0) µm Q = 1.35±0.02, surface rough, reddish orange to golden (6B8) when mature. *Paraphyses* hyaline, straight stick shape, 2.0–6 µm in diam, apical slightly inflated, septate, exceeding the asci by 180–300 µm.

**Plate 3.**
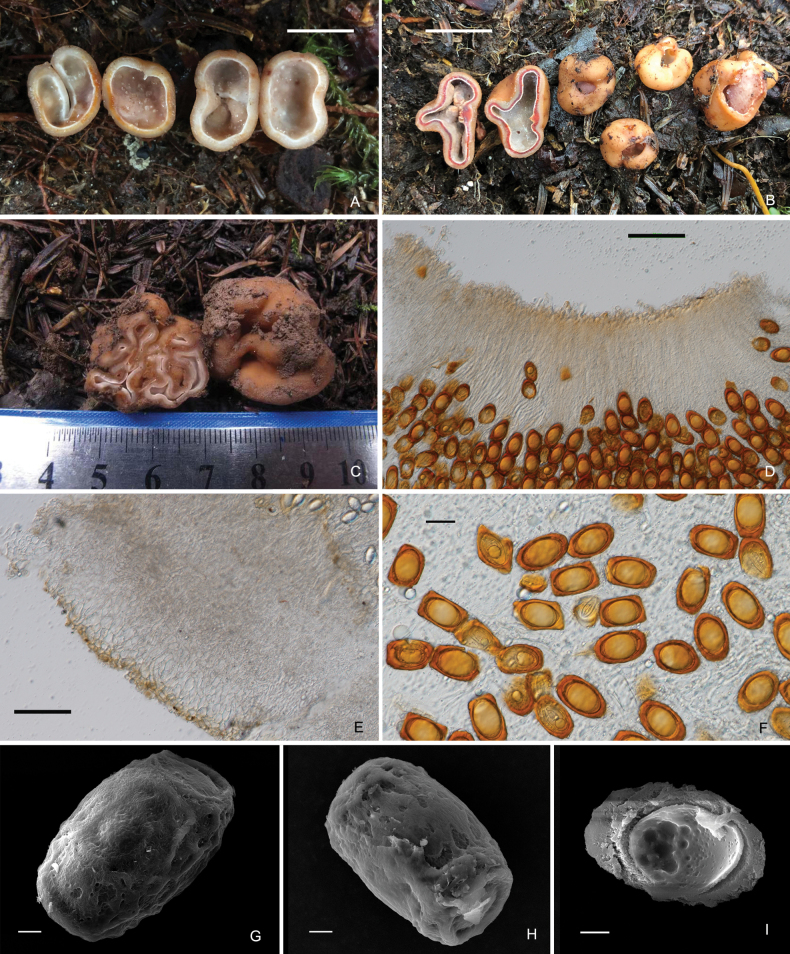
*Hydnotryalaojunshanensis***A** young sarcomata cut in half **B** mature ascomata with one cut in half **C** infolded and chambered ascoma **D** section of hymenium in 5% KOH **E** a peridium section in 5% KOH **F** ascospores released from asci in 5% KOH **G–I** ascospores under SEM (I. SEM of a single ascospore cut in half). Scale bars: 1 cm (**A, B**); 50 μm (**D, E**); 20 μm (**F**); 5 μm (**G–I**).

##### Ecology and distribution.

Hypogeous, solitary, or in groups in soil, under *Abies* spp., fruiting from late summer to early autumn. Known only from Yunnan Province, China.

##### Additional specimens examined.

China, Yunnan Province, Laojun mountains, 26°42.00'N, 99°42.00'E, alt. 3786 m, in a forest of A.forrestiivar.smithii, 30.Aug.2012, Lin Li (Holotype, YAAS L2425; GenBank KC878618); Shangri-La, 28°16.00'N, 99°11.00'E, alt. 3978 m, in a forest of *Abies* sp., 19 Aug. 2014, Shanping Wan (HKAS95802 GenBank: ITS = OP908303), Lijiang, 26°42.00'N, 99°58.00'E, alt. 3540 m, in a forest of *A.forrestii*, 12 Sept. 2019, Lin Li (BMDLU L21197 GenBank: ITS = ON982592, LSU = ON982620); Lijiang, 26°56.00'N, 99°32.00'E, alt. 3805 m, in a forest of *A.forrestii*, 21 Sept. 2021, Lin Li (BMDLU L21211 GenBank: ITS = ON982580, LSU = ON982621, BMDLU L21212 GenBank: ITS = ON982593, LSU = ON982622, BMDLU L21215 GenBank: ITS = ON982594, LSU = ON982623).

##### Notes.

When the species was described in 2013 by Li et al., only one collection from Mt. Laojun in Yunnan Province, China, was reported. More specimens of *H.laojunshanensis* have been found at other places in Yunnan since then. We discovered that this species had not only simple chambered ascomata but also folded, chambered ascomata. This species has large, rectangular ascospores (including thickened exsporium) with a rough surface differentiating from other species in *Hydnotrya*.

## ﻿Discussion

To date, 17 species of *Hydnotrya* (including these two new species) are accepted worldwide (Kirk et al. 2008; Stielow et al. 2010; Li et al.2013; Xu et al. 2018). The main macroscopic and microscopic characters of these species are provided and discussed based on available literature (Table 2).

**Table 2.** List of main characteristics of *Hydnotrya*.

Species	Ascomata	Gleba	Ascospore		Asci	Host Plants	Distribution	References
*Hydnotryabadia* L. Fan, Y.W. Wang & Y.Y. Xu 2018	Irregularly subglobose, 7–15 × 14–19 mm diam., surface even, brown to earth brown.	Gleba solid, with numerous variably compacted canals and chambers (usually without empty space).	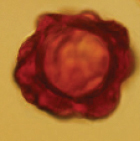	Roughly globose, 25–40 μm in diam. including ornamentation, 17.5–27.5 μm in diam. excluding ornamentation, red-brown to reddish, thickened exisporium with regular large protuberances	Asci broadly clavate to somewhat saccate, sessile, or narrowed at the base into a short stalk, 125–172.5 × 65–75 μm, randomly immersed in paraphyses, 8-spored, spores mostly biseriate.	*Pinus* sp.	Huize, China Asia 2000–2900m	Yu et al. 2018
*Hydnotryabailii* Soehner 1959	Irregularly subglobose, 10–20(–25) mm diam., dark brown, with deep furrows often with multiple lobes, with one or many openings at the apex, with pleasant aromatic smell.	Gleba solid, dark brown, strongly convoluted cavities.	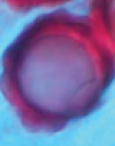	Globose, (27.5-) 30–34 (–37.5) μm in diam, brown reddish, exosporium thickening with blistered warts	Asci cylindrical, 250–300×30–40 μm, 8-spored, spores mostly uniseriate.	*Piceaabies*	Europe	Stielow et al. 2010
*Hydnotryabrunneospora* L. Fan, Y.W. Wang & Y.Y. Xu 2018	Irregularly globose, 20–23 mm diam., dark brown when dry, surface smooth.	Gleba solid, scattered with some small, isolated, and irregularly shaped chambers.	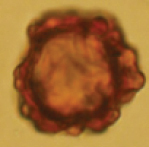	Roughly globose, 26.25–46.25 μm diam., brown to golden brown at maturity (never reddish), exosporium thickening with small protuberances.	Asci cylindrical to clavate, narrowed at the base into a short stalk, 162.5–237.5 × 30–47.5 μm, randomly immersed in paraphyses, 8-spored, spores mostly uniseriate.	*Betulaplatyphylla*	Jilin, China Asia	Yu et al. 2018
*Hydnotryacerebriformis* Harkn. 1899	Irregular spherical, lobulated, 10–35 × 10–20 mm diam., reddish-brown, cerebriform, with cavities that communicate with the gleba.	Gleba with labyrinthine chambers composed of invagination and fusion from the walls of the ascoma.	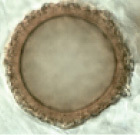	Globose ascospores 20–25μm diam. (x = 22.85 μm), excluding ornamentation, amber-brown, walls 1μm wide. Finely warty ornamentation, warts up to 4μm long.	Asci cylindrical, 175–200 × 25–35 μm, 8-spored, spores mostly uniseriate.	*Pinus* sp. *Abies* sp.	Europe North America 3100–4000m	Harkness 1899 Abbott and Currah 1997 Piña-Páez et al. 2017
*Hydnotryaconfusa* Spooner 1992	Ovoid or irregular, size from ca. 20 × 20 × 15 cm up to 40 × 40 × 20 mm, greyish-brown or red-brown, with a primary apical opening and sometimes some smaller secondary openings.	Gleba hollow, with single chambered but mostly cerebriform folded.	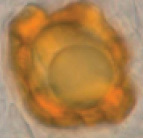	Ovoid or very broad ellipsoid, 38–50 × 28–32 µm, golden brown, exosporium much thickened, vertically grooved, forming irregular warts.	Asci cylindrical, 290–320 × 38–43 μm, 8-spored, spores mostly uniseriate. clavate at immature, with irregular or biseriate, cylindric at maturity, strictly uniseriate.	*Picea* sp.	Europe 361m	Spooner 1992 Bemmann and Bandini 2011
*Hydnotryacubispora* (E.A. Bessey & B.E. Thomps.) Gilkey 1939	Irregularly globose, 5–10 mm diam., Isabella color, with somewhat cerebriform folds radiating distinctly from central opening	Gleba with cavity simple, but somewhat irregular due to surface lobing.	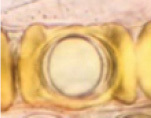	Cubical, 47–50 × 23–32µm, including thickening exosporium, brownish, with maturity.	Asci cylindrical, 100–120 μm long, 8-spored, spores mostly uniseriate.	Coniferous forest	Europe North America	[Bibr B11][Bibr B6] K(M)189248
*Hydnotryainordinat*a Trappe & Castellano, 2000	Irregular globose, 8–30mm diam., dark red-brown, convolute and infolded ptychothecia with one or a few openings from the interior	Gleba complex, of infolded tramal plates forming canals and chambers 0.5–3mm broad.	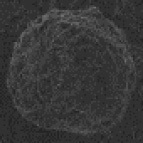	Globose to ellipsoid, 20–30×20–28μm excluding ornamentation, brown-yellow, with aggregated , irregular flexuous spines	Asci cylindrical, ±300 × 25–33 μm, (6–) 8-spored, spores mostly uniseriate.	*Abiesamabilis Tsuga mertensiana*	North America 1800m	Trappe and Castellano 2000
***Hydnotryalaojunshanensis*** Lin Li, D.Q. Zhou & Y.C. Zhao 2013	Irregularly globose, 10–30 mm diam., brownish orange, smooth, mostly single-chambered with a primary apical opening, rare the opening narrowing into a slit, sometimes folded forming a few channels, lined with white fluffy hymenium. No special smell.	Gleba hollow, single-chambered, sometimes infolded and chambered, lined with hymenium with orange asci and whitish to yellowish paraphyses.	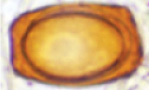 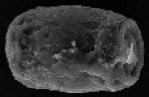	Ellipsoid without thickened exosporium, rectangle (including exosporium), (42.5–) 50.0–57.2(–60.3) × (27.5–)30.4–36.9(–38.2) µm, reddish orange, thickening exosporium with rough surface.	Asci cylindrical, 331.5–390.5 × 25.5–35.5 µm, 8-spored, spores strictly uniseriate	*Abies* spp.	Yunnan, China Asia 3500–3800m	Li et al. 2013 This study
*Hydnotryamichaelis* (E. Fisch.) Trappe 1975	Irregular or subspherical, up to 60 mm across, with rounded opening, wrinkled, lobulate, with numerous invaginations, odor very strong, somewhat pungent, rather persistent.	Gleba labyrinthoid, with large, sinuous cavities, separated by folded inwards portions of ascoma wall.	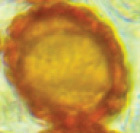	Broadly ellipsoid, (21.2–)24.9–29.6(–32.2) ×(18.8–)19.8–22.4 (–24.9) µm; ornamentation excluded), honey-yellow, exosporium thickened, with conspicuous, irregular, often interconnected warts	Asci cylindrical, 200–220 × 30–35 µm, 8-spored, spores strictly uniseriate	Pinaceae	Europe North America	Trappe 1975 Slavova et al. 2021
*Hydnotryanigricans* L. Fan, Y.W. Wang & Y.Y. Xu 2018	Irregular globose, 13 × 9mm, black brown to blackish	Gleba solid, brown, red to dark reddish, with some irregularly shaped and isolated small chambers lined with pale whitish hymenium.	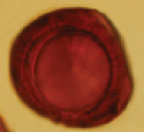	Irregularly globose, 25.0–37.5 μm in diam., red brown, exosporium unevenly thickened, and usually of trigonal outline in cross section	Asci broadly clavate to saccate, sessile or narrowed at the base into a short stalk, 87.5–190 × 25–62.5 μm, scattered between paraphyses in a hymenium,8-spored,with spores mostly biseriate.	*Pinus* sp.	Sichuan, China Asia	Yu et al. 2018
***Hydnotryaoblongispora sp. nov.***	Irregularly globose, 10–25mm in diam. when fresh, light khaki to reddish brown, smooth, mostly single-chambered with a primary apical opening up to 02–08 mm in diam., sometimes infolded.	Gleba hollow, single-chambered lined with milky white hymenium, hymenium surface fluffy.	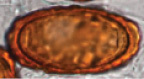 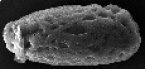	Long-ellipsoid (20.0–) 26.5–39.0 × (9.5–) 11.0–21.5 μm, golden brown, thickened exporium with pitted surface.	Asci cylindrical, 102.5–138.5 × 13.0–25.5 µm, narrowed at the base into a long stalk (20–30 μm), 8-spored, spores strictly uniseriate	*Abiesforrestii*	Yunnan, China Asia 3500–4000m	This study
*Hydnotryapuberula* L. Fan, Y.W. Wang & Y.Y. Xu 2018	Irregularly subglobose, 11–20 × 8–19 mm, brown to dark brown, sometimes with purple tints when fresh, much convoluted with deep furrows, ascoma surface tomentulose	Gleba solid, compact, dark brown to purple reddish at maturity, with numerous small chambers.	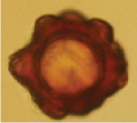	Roughly globose, 22.5–42.5 μm in diam., red brown to reddish, exosporium unevenly thickened by irregularly large protuberances.	Asci clavate to saccate, 125–190 × 55–80 μm, sessile or with a short stalk, borne among palisade-like paraphyses in the hymenium, 8-spored, with spores mostly biseriate.	*Pinus* sp.	Yunnan, China Asia	Yu et al. 2018
*Hydnotryasoehneri* Svrček, 1955	Irregularly subglobose, tuberous, 10–40 mm wide, reddish and reddish-gray to reddish brown, odor light fragrance	Gleba solid, whitish to yellowish gray, at maturity is colored reddish-brown corridors (from mature spores).	Spherical, 25 – 36 (– 42), red brown, exosporium thickened, coarsely warty.		Asci mostly cylindrical to saccate, 150–300 × 35–70 µm, 8-spored, mostly incompletely arranged biseriate.	Mixed woods	Europe	Svrček 1955
*Hydnotryasubnix* Trappe & Castellano, 2000	Irregular subglobose, 50–65mm in diam, dark red-brown, glabrous to minutely roughened. Odor and taste strongly of spicy garlic.	Gleba variable, deeply convoluted and infolded lacking openings from the interior, forming canals and locules 1–10mm broad.	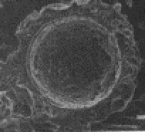	Globose to rarely ellipsoid, 23–30μm in diam. excluding ornamentation, brown, coarsely warty.	Asci mostly cylindrical, 300–340 × 25–40 µm, 8-spored, mostly incompletely arranged uniseriate	*Abiesamabilis*	North America 950m	Trappe and Castellano 2000
*Hydnotryatulasnei* (Berk.) Berk. & Broome, 1846	Irregularly spherical or lobed, sometimes with inward folds, 20–70 mm diam., ochre-reddish to brick red	Gleba solid, later yellow brown, with labyrinthic chambers.	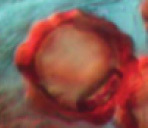	Globose, 20–30 (–33) μm diam. (including ornamentation), ochre-reddish, with conspicuous, irregular warts.	Asci broadly clavate or cylindrical, 175–210 × 30–62.5 µm, (4–) 8-spored, spores biseriate.	Coniferous forest	Europe North America 1600m	Dimitrova and Gyosheva 2008 Stielow et al. 2010
*Hydnotryavariiformis* Gilkey, 1947	Globose to subglobose to flattened, somewhat depressed, 7–40 mm broad, cinnamon-buff to cream-buff	Gleba variable, from a simple cavity to extremely lobed with numerous small chambers the interior, usually opening to the exterior at one or more points.	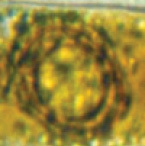	Ellipsoid, 24–28×36–36 μm, yellow-brown, thickened exosporium wall, surface appearing punctate and with small irregular nodules	Asci 240–280 × 24 µm, 8-spored, clavate at immaturity, spores incompletely biseriate; cylindrical at maturity, spores strictly uniseriate.	Coniferous forest	North America 1200–2400m	Gilkey 1947 Abbott and Currah 1997 Beug et al. 2014
***Hydnotryazayuensis sp. nov.***	Irregularly globose, 15–20 mm in diameter when fresh, smooth, gentle inward folds, surface cinnamon. Mostly single-chambered with a primary apical opening, the opening is just an almost closed seam, white fluffy inside cavity. Elastic and crisp. No special smell.	Gleba hollow, single-chambered with a primary apical opening, sometimes the opening is just an almost closed seam.	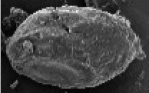 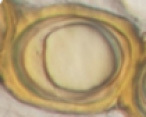	Ellipsoid, (17–)20–30.5 × 15.5–18.0 μm, (including thickened exosporium), golden yellow, surface rough, looking like crumbly.	Asci cylindrical, 118.5–130.5×15.0–22.5 µm, 8-spored, spores strictly uniseriate	*Abies* sp.	Zayu, China Asia 3770m	This study

The ascospore morphology is highly variable among different species in *Hydnotrya*, which is useful for distinguishing species. Abbott and Currah (1997) once divided the genus *Hydnotrya* into two subgenera: subg. Hydnotrya and *Cerebriformae*, according to the characters of their ornamentation. The subg. Hydnotrya had four species of *H.tulasnei*, *H.michaelis*, *H.cubispora*, and *H.variiformis* showing ascospores with rounded or irregular warts. The subg. Cerebriformae has only one species of *H.cerebriformis* differs from Subg. Hydnotrya in ascospores with short, rounded aculei. However, the current phylogenetic analysis showed that ascospore characteristics were not reliable for differentiating species of *Hydnotrya* into these subgenera (Fig. 1).

Based on ITS analyses, 14 species of *Hydnotrya* are divided into two lineages, A and B. The species in the clade A mostly have nearly solid gleba (6 out of 9) and globose, warty ascospores, either uniseriately or biseriately arranged in asci. The clade A is divided into two subclades: the subclade Aa (clade 1–6) and Ab(clade 7–9). The species in the subclade Aa have solid ascomata. Two groups can be distinguished: the group 1 (clade 1 and 2) and group 2 (clade 3–6), both found in China and Europe. The group 1 contains two species with ascospores uniseriately arranged in asci; the group 2 contains four species with ascospores biseriately arranged in asci. Species in the subclade Ab are distributed in China, Europe, and America, and have hollow ascomata and ascospores uniseriately arranged in asci. The species in the clade B has hollow to chambered gleba and ellipsoidal ascospores (without thickened exosporium), biseriately arranged in asci. The clade B is divided into two groups: Ba and Bb. The group Ba (clade 10 and 11) contains 2 species distributed in China and Europe, with ellipsoidal ascospores, with a pitted surface. The group Bb (clade 13 and 14) contains two species, only found in China, with rectangular and ellipsoidal ascospores (with thickened exosporium), with a rough surface. (Fig. 1).

Based on the morphological and molecular phylogenetic analyses there seems to be a trend in morphological traits among the species within the genus *Hydnotrya*, that is, the gleba evolved from being hollow or chambered to nearly solid; the ascus becoming shorter and wider, with ascospores arranged from uniseriate to biseriate; ascospores from ellipsoidal to globose, with an ornamentation from smooth to rough as well. This evolutionary trend in the genus *Hydnotrya* is probably related to their hypogeous habits, that is, if the gleba has more chambers, the ascoma will hold more ascospores, and so there are more chances of ascospores to be dispersed by animals that eat them (Hawker 1955; Ławrynowicz 1990; Læssøe and Hansen 2007; Bonito et al. 2013). All of this improves their survival and reproduction. Of course, more collections would be needed for comprehensive morphological and molecular analyses to provide more evidence to support this hypothesis.

In China, 9 species were recorded before this study (Xu et al. 2018). In this paper, two new species are described. 11 species are now known in China, among which 7 species are distributed in southwest China.

### ﻿Key to species of *Hydnotrya*

**Table:** 

1	Ascomata hollow, gleba chamber simple or infolded	**2**
–	Ascomata solid, gleba labyrinthine chambered	**11**
2	Ascospores rectangular or cubical	**3**
–	Ascospores ellipsoidal or globose	**4**
3	Ascospores cubical	***H.cubispora***
–	Ascospores rectangular	***H.laojunshanensis***
4	Odor distinct, with a special smell	**5**
–	Odor not distinct	**6**
5	Odor and taste strongly garlic	***H.subnix***
–	Odor strong pungent and persistent	***H.michaelis***
6	Ascospores mostly globose	**7**
–	Ascospores ellipsoidal or long ellipsoidal	**8**
7	Ascospores globose, with prominent echinate ornamentation	***H.cerebriformis***
–	Ascospore mostly globose, with aggregated, irregular flexuous spines	***H.inordinata***
8	Ascospores long ellipsoidal, surface pitted, ascomata mostly single chambered	***H.oblongispora***
–	Ascospores ellipsoidal, Q ratio less than 2	**9**
9	Ascospores incompletely biseriate at immaturity, strictly uniseriate at maturity in asci	**10**
–	Ascospores strictly uniseriate from immature to mature asci	***H.zayuensis***
10	Ascospores broadly ellipsoidal, vertically grooved, forming irregular warts	***H.confusa***
–	Ascospores ellipsoidal, surface appearing punctate and with small irregular nodules	***H.variiformis***
11	Ascospores mostly uniseriate	**12**
–	Ascospores mostly biseriate	**13**
12	Ascospores less than 35 μm^1^ in length, reddish brown	***H.bailii***
–	Ascospores up to 46 μm^*^ in length, brown to golden brown	***H.brunneospora***
13	Odor with a light fragrance	***H.soehneri***
–	Odor not distinct	**14**
14	Ascoma surface tomentose, withpurple tints when fresh	***H.puberula***
–	Ascoma not tomentose	**15**
15	Ascospores without prominent protuberances, trigonal outline in cross section, ascomata blackish	***H.nigricans***
–	Ascospores with recognizable protuberances	**16**
16	Ascospores, 20–30 μm diam.^*^, ochre-reddish, with conspicuous, irregular warts	***H.tulasnei***
–	Ascospores, 25–40 μm in diam.^*^, red brown to reddish, with regular large protuberances	***H.badia***

## Supplementary Material

XML Treatment for Hydnotrya oblongispora

XML Treatment for Hydnotrya zayuensis

XML Treatment for Hydnotrya laojunshanensis

## Footnotes

Including ornamentation.

## ﻿Additional information

### Author ORCIDs

Lin Li https://orcid.org/0009-0000-8167-2965

Shan-Ping Wan https://orcid.org/0000-0002-0794-3701

Naritsada Thongklang https://orcid.org/0000-0001-9337-5001

Song-Ming Tang https://orcid.org/0000-0002-6174-7314

Zong-Long Luo https://orcid.org/0000-0001-7307-4885

Shu-Hong Li https://orcid.org/0000-0001-5806-9148

### Data availability

All of the data that support the findings of this study are available in the main text.
